# A novel fully tapered, self-cutting tissue-level implant: non-inferiority study in minipigs

**DOI:** 10.1007/s00784-021-03912-w

**Published:** 2021-04-16

**Authors:** Edgard El Chaar, Algirdas Puisys, Itai Sabbag, Benjamin Bellón, Aikaterini Georgantza, Wayne Kye, Benjamin E. Pippenger

**Affiliations:** 1 Department of Periodontology and Implant Dentistry, New York University, New York, USA; 2 Vilnius Research Group, Vilnius, Lithuania; 3 Vilnius Implantology Center, Vilnius, Lithuania; 4 Lahav Research Institute, Kibbutz Lahav, Israel; 5 Department of Preclinical and Translational Research, Institut Straumann AG, Basel, Switzerland; 6 Department of Periodontology, Faculty of Dentistry, University of Zurich, Zurich, Switzerland; 7 Department of Periodontology, Faculty of Dentistry, University of Bern, Freiburgstrasse 7, CH-3010 Bern, Switzerland

**Keywords:** Osseointegration, Fully tapered, Self-cutting, Tissue level, Implant

## Abstract

**Objectives:**

To assess the osseointegration and crestal bone level maintenance of a novel fully tapered self-cutting tissue-level implant for immediate placement (test) compared to a clinically established tissue-level implant (control) in moderate bone quality.

**Materials and methods:**

Test and control implants were compared in 3 groups, i.e., small-, medium-, and large-diameter implants in an edentulous mandibular minipig model with moderate bone quality after 12 weeks of healing. Histometrically derived bone-to-implant contact (BIC) and first bone-to-implant contact (fBIC) were subjected to statistical non-inferiority testing. Maximum insertion torque values in artificial bone were assessed for comparison.

**Results:**

BIC values for the tests and control implants for all 3 diameters were comparable and non-inferior: small diameter (61.30 ± 10.63% vs. 54.46 ± 18.31%) (*p*=0.99), medium diameter (60.91 ± 14.42 vs. 54.68 ± 9.16) (*p*=0.55), and large diameter (45.60 ± 14.67 vs. 52.52 ± 14.76) (*p*=0.31). fBIC values for test implants were higher and non-inferior compared to control implants in all three groups. Test implants further showed distinctly higher maximum insertion torque values compared to control implants.

**Conclusion:**

The investigated novel tissue-level implant is able to achieve high levels of primary and secondary implant stability under simultaneous preservation of crestal bone levels. This qualifies the studied implant as an attractive candidate for immediate placement in bone of limited quality.

**Clinical relevance:**

This pilot pre-clinical study investigated a novel tissue-level implant for immediate placement. With the aim of translating the studied prototype into clinical application pre-clinical models, procedures and controls have been chosen with the aim of reflecting its future clinical indication and use.

**Supplementary Information:**

The online version contains supplementary material available at 10.1007/s00784-021-03912-w.

## Introduction

Tissue-level implants represent one of the earliest and well-established treatment concepts in implant dentistry. The concept emerged in the early 1970s and was adopted for routine rehabilitation of fully or partially edentulous patients since the mid-1980s. Compared to submerged treatment concepts, tissue-level implants require a reduced number of surgical procedures and chair-time for the patient [1]. Although bone level implants may potentially provide higher prosthetic flexibility, tissue-level implants still represent a treatment concept which can be considered equivalent in terms of osseointegration, better in crestal bone level preservation and equivalent with regards to long-term survival rates [2, 3]. The presence of the microgap at bone level has been implicated as a potential source for bacterial colonization and mechanical micromovements that lead to crestal bone resorption. A key characteristic that may have contributed to the long-term clinical success of tissue-level implants and the crestal bone preservation is the relocation of this microgap away from the bone level [4–6].

Implant dentistry is trending towards early and immediate procedures, outperforming the number of late implant placement procedures [7]. Other attempts focus on the extension of established treatment concepts into patient populations with compromised pre-conditions and reduced bone quality or limited bone quantity [7–9]. These trends require constantly readapt the design principles of dental implants and surgical workflows to meet the biomechanical and biological requirements under such conditions [10–12]. Specifically, primary implant stability has emerged as one of the key criteria in this context [13]. Optimal primary implant stability is considered as a pre-requisite for the establishment of osseointegration and is therefore considered as indicative for the prognosis of implant success [14, 15]. Its direct routine assessment as part of clinical procedures by means of resonance frequency analysis or insertion torque measurements is well established [11]. Tapered implants have evolved as prominent candidates to improve primary stability and a multitude of individual tapered implant designs have been proposed [10, 16]. The optimization of such designs often requires careful consideration and fine-tuning of several design features like, e.g., the implant core dimensions, thread geometry, and the specific design of optional integrated cutting elements in parallel, as well as surgical preparation techniques of the implant osteotomy [10].

Recently, a new self-cutting tapered implant design has been commercially introduced that includes specific design features to maximize primary stability in situations with decreased bone quality. The key characteristic in this implant design is a protruding thread geometry, which allows the surrounding bone to be condensed and at the same time, limits excessive stress specifically on the cortical bone by a process termed active bone management [17]. Within this pre-clinical study, we have evaluated a further extension of this design concept into a fully tapered self-cutting tissue-level implant for immediate placement. The specific aim of this non-randomized controlled in vivo pilot study was to assess and compare the osseointegrative properties and crestal bone level maintenance around this novel type of tapered implant in comparison to a clinically long-term established tissue-level implant as benchmark in a situation with moderate bone quality. Insertion torque measurements in model substrates were further performed to evaluate and compare the primary stability of both tested implant systems in the context of the in vivo performance.

## Materials and methods

The primary aim of this non-randomized controlled pre-clinical pilot study was to compare the osseointegration of a novel fully tapered, self-cutting tissue-level implant (test device; Straumann® TLX, Straumann, Basel, Switzerland) to a clinically long-term established tissue-level implant (control device; Straumann® Standard Plus, Straumann, Basel, Switzerland) as part of a non-inferiority study design. A mandibular minipig model using a single end point at 12 weeks after implantation was chosen as a test system. Test and control implants were compared for three different implant diameters (study groups), i.e., small, medium, and large diameters. Specifications of the specific implants per group are provided in Table 1. A total of 48 implants (8 implants per group and animal) were placed in fully healed edentulous mandibles (3 implants per hemi-mandible) of Sinclair Minipigs^TM^ in a split-mouth arrangement, i.e., each minipig received small-, medium-, and large-diameter implants of test and control on each side. In order to mitigate potential effects of implant position on histological outcomes, implantation sites of individual implant types were allocated across the mesial-distal axis and side switched from animal to animal such that each implant was placed in each anatomical site at least once. Primary and secondary parameters of the study were the histometrically derived percentage of bone-to-implant contact (BIC) and first bone-to-implant contact (fBIC) as schematically illustrated in Fig. 2b. Parallel laboratory tests compared the primary stability of the implants types in terms of insertion torques in polyurethane plates.

**Table 1:** Comparison of implant design characteristics for test and control implants per implant group

Group	Item	Implant design characteristics					Osteotomy
		Endosseal design	Supracrestal design	Neck length ^†^ (mm)	Thread diameter (*Ø*) (mm)	Core diameter (mm)	Final drill diameter (mm)
Small diameter	Test	Slim core, fully tapered	WN, *Ø*4.8	1.65	3.75	3.5	3.2/3.7
	Control	Parallel wall	RN, *Ø*4.8	1.8	4.1	3.5	4.1 (profile)
Medium diameter	Test	Slim core, fully tapered	WN, *Ø*4.8	1.65	4.5	3.5	3.7
	Control	Parallel wall	RN, *Ø*4.8	1.8	4.8	4.2	4.8 (profile)
Large diameter	Test	Slim core, fully tapered	WN, *Ø*6.5	1.45	5.5	4.5	4.7
	Control	Parallel wall	WN, *Ø*6.5	1.8	4.8	4.2	4.8 (profile)

^†^The neck length is defined as vertical distance between the rough endosseal surface margin and the horizontal plane of the supracrestal part displaying the maximum diameter

This study was conducted at Lahav C.R.O, Lahav, Israel, in accordance with the Israeli National Council of Animal Experimentation. This study adhered to the ARRIVE Guidelines and was designed and performed under consideration of the 3R (Replace, Reduce, Refine) guidelines for animal experimentation.

Eight (8) female Sinclair Minipigs™ (Meshek ben meir, Israel) of age between 20 and 24 months at time of surgery and with a bodyweight of 40 to 60 kg were included in the study (3 implants/hemi-mandible; 6 implants per animal; a total of 48 implants). The animals were housed in standard boxes in groups of 2 to 4 animals. Standard diet (soft food) expanded for Minipigs from AMBAR Feed mill (Granot M.P. Heffer 3881100, Israel) was provided. Housing started at least 10 days prior to intervention to adapt the animal to the experimental environment. All animals were fasted overnight prior to the surgical procedures to prevent vomiting.

### Surgical procedure

All surgical procedures were performed under aseptic conditions in an operating suite dedicated to veterinary surgery and under general anesthesia: Prior to the surgeries, 2 mg/kg xylazine (Anased®, AKORN, USA) + 10 mg/kg ketamine (Clorkeam®, Vetoquinol, France) were administered by intramuscular injection. Anesthesia was induced by inhalation of 3% isoflurane (Piramal Critical Care, USA) via a mask and intravenous administration of 5 to 10 mg diazepam per animal (diazepam, Ratiopharm®, Ratiopharm, Germany). During surgeries, anesthesia was maintained by the administration of 1–3% isoflurane. Animals were intubated and breathing was withheld by a ventilator. Vital parameters were monitored continuously (pulse oximetry, rectal temperature, blood pressure, CO2). To reduce the amount of systemic analgesic and bleeding during surgery, local anesthesia with 2.5 ml lidocaine/adrenaline (lidocaine 20 mg/ml, Esracain®, Rafa lab, Israel + adrenaline 1 mg/ml, Adrenalina®, Galenica senese, Italy) was provided intraoperatively. Post-operative pain relief was managed by intramuscular injection of 0.1 mg/kg buprenorphine (Buprenorphine®, vetmarket, Israel) for 2–4 days as judged by the veterinarian. Antibiotic prophylaxis was administered using 0.1 ml/kg Pen&strep® (200 mg/ml penicillin + 250 mg/ml streptomycin, Norbrook, USA) once a day for 3 days post-operatively. Within the first days after surgery (healing phase), the animals were monitored routinely and further analgesia was given as necessary.

### Tooth extraction

Preparation of test sites was based on careful extraction of contralateral mandibular premolars (P2–P4) and first mandibular molars (M1) under general anesthesia via a minimally invasive surgical approach, i.e., without raising a flap.

### Implant osteotomy and placement

**Fig. 1:**
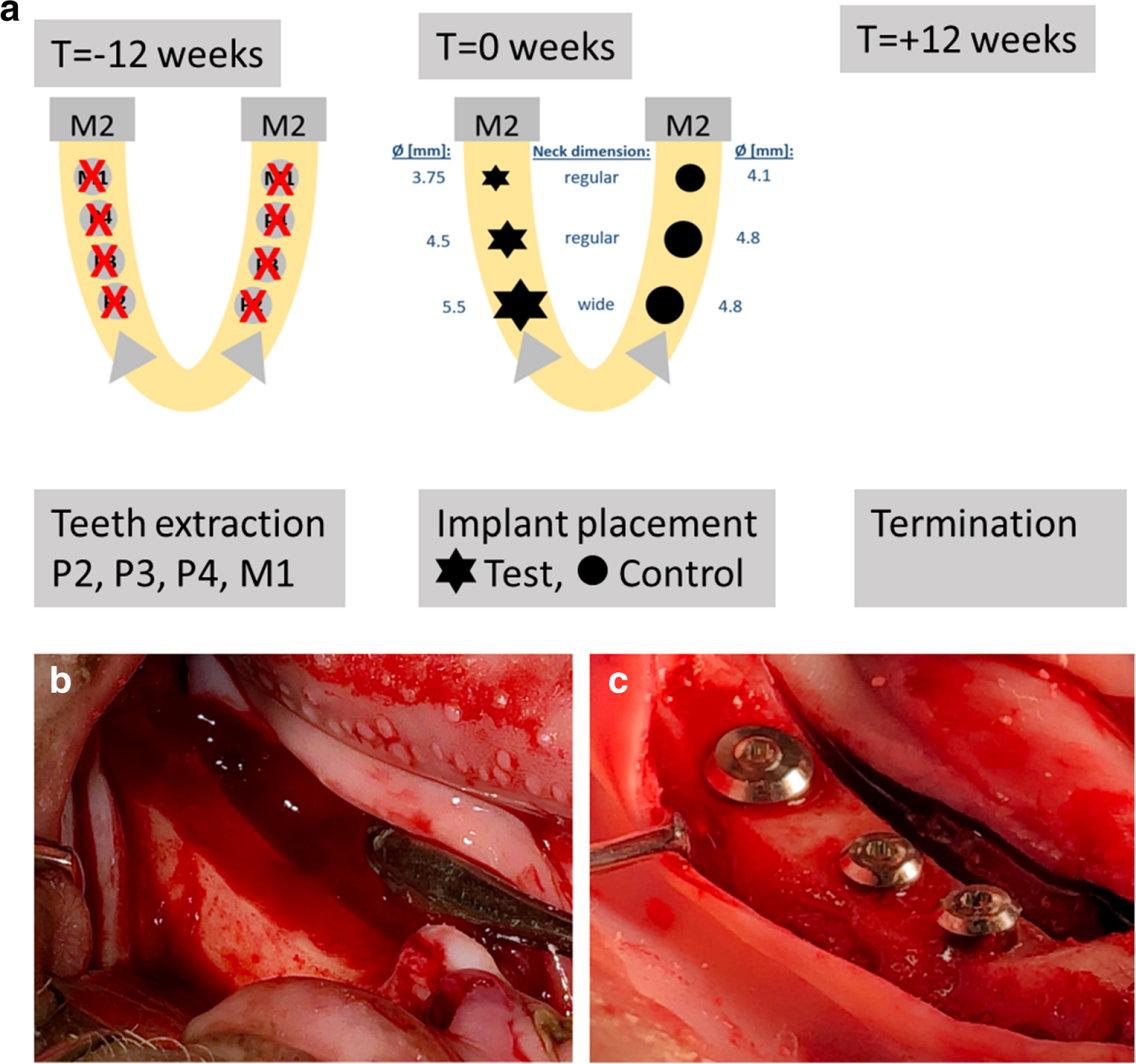
Overview of the surgical timeline and implantation scheme (**a**) and representative photographs illustrating the main steps of the surgical procedure. **b** Prepared implantation site after a full-thickness muco-periostal flap preparation and flattening of the alveolar bone crest. **c** Implantation site after osteotomy preparation, implant placement, and installation of healing caps before primary wound closure. The image shows test implants with diameters *Ø*3.75 mm (posterior), *Ø*4.5 mm (middle), and *Ø*5.5 mm (anterior)

Test and control implants were placed 12 weeks post extraction by the scheme as illustrated in Fig. 1. Procedures were initiated by exposing both sides of the mandibular alveolar ridge via a mid-crestal incision and reflecting a full-thickness muco-periosteal flap. The alveolar ridge was carefully flattened using a cylindrical cutting bur (Fig. 1b). Implant osteotomies for the control implants were prepared according to the manufacturer’s instruction using pilot drills and Twist drill PRO drills (Straumann, Switzerland). Specifically, the following sequences of combinations of drill diameters and rotational speeds were used for small-diameter control implants: *Ø*2.2 mm, 800 rpm → *Ø*2.8 mm, 600 rpm → *Ø*3.5 mm, 500 rpm, followed by tapping and profiling. Medium- and large-diameter control implants included use of *Ø*4.2-mm drills at 400 rpm. Osteotomies of test implants were prepared using the VeloDrills drill system (Straumann, Switzerland). According to the manufacturer’s instructions, specific drill sequences were adapted intraoperatively as judged by the operating surgeon. Final drill diameters for medium- and large-diameter test implants were *Ø*3.7 mm and *Ø*4.7 mm, respectively. Osteotomies for the small-diameter test implants used a combination of *Ø*3.2-mm drills for the apical half and in addition *Ø*3.7-mm drills for the preparation of the crestal half of the osteotomy (see Table 1). All implants were placed with the junction between the rough and smooth surface at crestal bone level using a motorized hand piece. Implants were subsequently equipped with healing caps (titanium cover screws, 0 mm in height) before primary wound closure of wound margins for submerged healing using resorbable sutures.

Antibiotic cover was administered for 3 days post-surgery (Pen&strep, Norbrook, UK, 1 ml/10 kg IM). Analgesics were administered if considered necessary as described above.

### Termination

Termination was performed at 12 weeks post-implantation. Specifically, intra-cardiac arrest was induced by injecting a 20% solution of pentobarbital (Pental Veterinary, CTS Chemical Industries, Israel).

Block section of the mandibular implantation sites was prepared using an oscillating autopsy saw under perseveration of the soft tissues. Sections were fixed in formalin (4% solution formaldehyde solution) for at least 2 weeks prior to histological processing.

### Histological processing

Block sections were immersed in formalin buffer solution, dehydrated using ascending grades of alcohol and xylene, and subsequently infiltrated and embedded in methyl methacrylate for non-decalcified sectioning. Block sections were next cut in bucco-lingual direction to sections of 500 μm and ground to a final thickness of 30–50 μm before staining them with paragon (toluidine blue and basic fuchsine) for microscopic evaluation.

### Quantitative histomorphometry

**Fig. 2:**
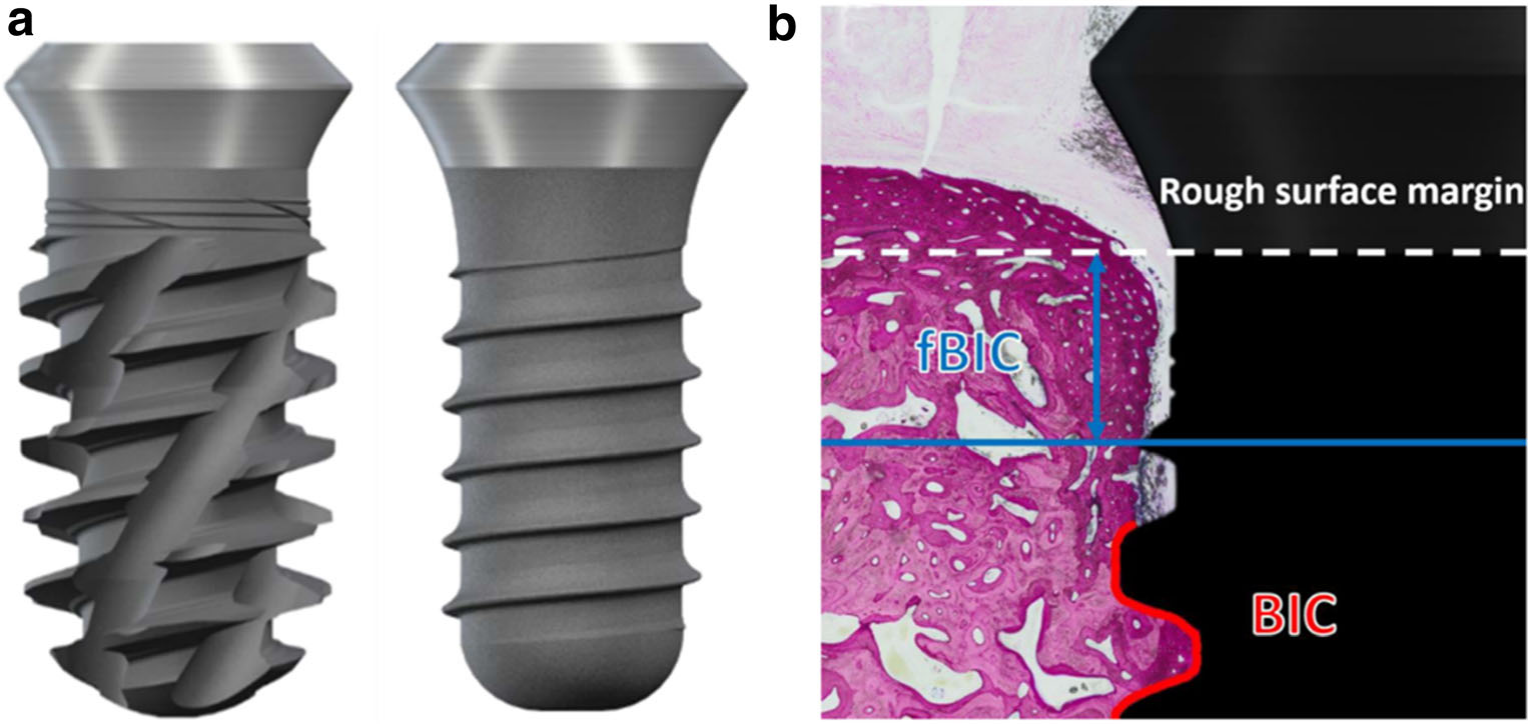
**a** Side-by-side comparison of schematic representations of test (left) and control (right) implants. **b** Illustration of histometric derived values, i.e., bone-to-implant contact (BIC) and first bone-to-implant contact (fBIC). BIC was evaluated as percentage of the perimeter of the endosseal part of the implant in contact with bone. First bone-to-implant contact (fBIC) was evaluated as the minimum distance from the rough surface margin of the implant to the apical bone-to-implant contact (blue arrow)

Histometric measurements were performed on central bucco-lingual sections of the implant. Histometric parameters comprised the bone-to-implant contact (BIC) as defined by the relative percentage of the perimeter of the endosseal part of the implant in contact with bone and the first bone-to-implant contact (fBIC) as defined by the distance between the rough-to-smooth surface margin of the implant and the most apical level of crestal bone in contact with the implant surface. fBIC values were reported as averages of values derived on the buccal and lingual aspects allowing positive and negative values resulting from crestal bone levels lying more coronal or apical with regards to the reference margin of the implant respectively (Fig. 2b).

### Insertion torques and primary stability tests

Simulation of implant insertion and measurements of insertion torques were carried out as previously reported in a bench test [11]. Implant types and dimensions as well as osteotomy preparation and implant insertion protocols were identical to the ones used for the in vivo tests. Solid rigid polyurethane (PUR) foam blocks (Sawbones Europe, Malmoe, Sweden) with a bone density of 30 pcf/0.48 gcm^−3^ corresponding to type 2 bone were used. Ten small- and medium-diameter implants and 12 large-diameter implants of each type were placed in a standardized procedure. Maximum insertion torques were derived from torque vs. insertion plots that were recorded using a torque testing machine adapted for perpendicular implant positioning (Walter+Bai, Loehningen, Switzerland). Values are reported as averages and standard deviations. Insertion torque values of individual groups were not compared using statistical tests.

### Statistical evaluation

The total number of implants included in the analysis was 47. One implant (a TL *Ø*4.1 RN) was not placed due to limited anatomical space in the mandible of one animal. The P3 of this animal was impacted and it was decided intraoperatively to drop 1 implant from the TL group. Values for measured parameters were summarized as means, standard deviations, medians, and interquartile ranges. Test and control samples of individual groups (small-, medium-, and large-diameter implants) were compared using the Wilcoxon signed-rank tests.

Adjusted comparisons and non-inferiority tests were performed using a mixed linear regression model that adjusted for animal effect, side of the mandible, and position of test and control implants. The animal effect was introduced in the model as a random effect. All other factors were set as fixed effects. The Dunnett-Hsu adjustment was used to adjust the *p* values in the case of multiple comparisons. A *p* value of <0.05 was considered statistically significant. For the hypothesis of non-inferiority of the test device compared to a control device, the average effect and its two-tailed 90% confidence interval (equivalent to a one-tailed 95% confidence interval) were calculated. The lower limit of the confidence interval served as the tolerance range (TR) for the support of the null hypothesis. A detailed description of non-inferiority testing is provided as part of the supplementary part.

Non-inferiority testing of BIC between implants of specific subgroups was performed on the basis of the following null and alternative hypotheses (*H*_0_ and *H*_1_):

- *H*_0_: Average BIC (test) ≤ Average BIC (control)−TR
- *H*_1_: Average BIC (test) > Average BIC (control)−TR

Non-inferiority testing of fBIC between implants of specific subgroups was performed with the following null and alternative hypotheses (*H*_0_ and *H*_A_):

- *H*_0_: Average fBIC (test) ≤ Average fBIC (control)−TR
- *H*_1_: Average fBIC (test) > Average BIC (control)−TR

## Results

All animals recovered from surgeries in a predictable and uneventful manner. No specific surgical, peri- or post-operative complications or signs of inflammation were registered. Control group data for the small-diameter implants was reduced to *n*=7 due to limited space of the edentulous implantation site in one animal noted during surgery. All other test and control implants were placed and evaluated as planned with *n*=8 implants per study group.

### Histologic and histometric analysis

**Fig. 3:**
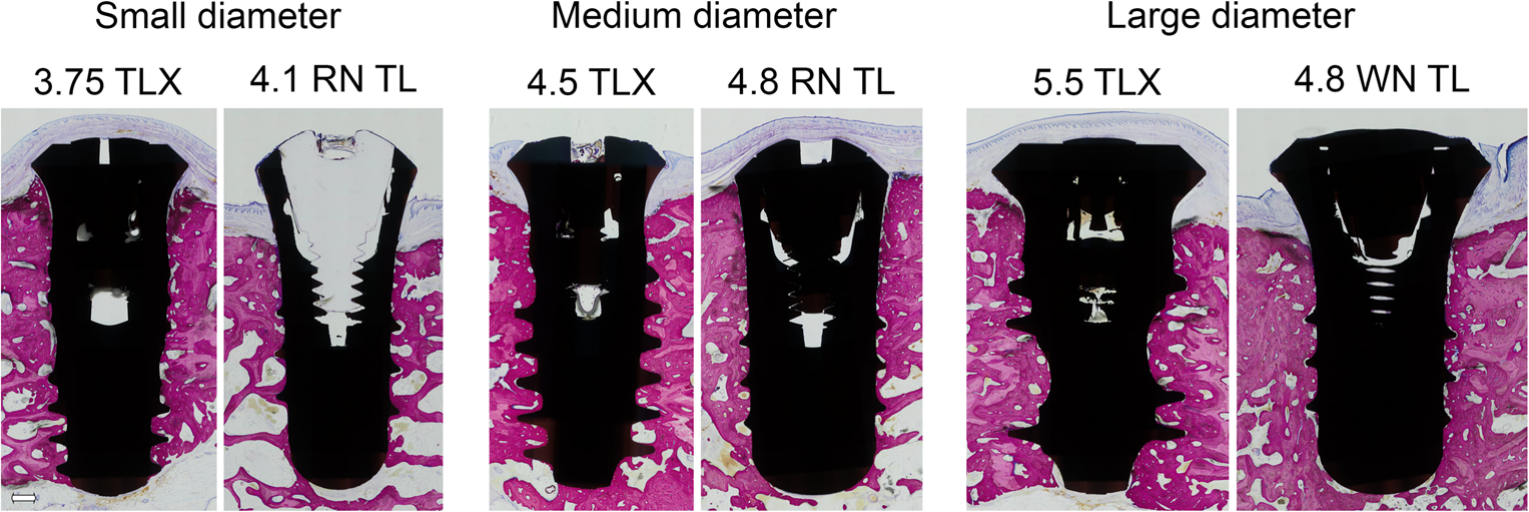
Representative bucco-lingual histological sections of each test and control implant

As illustrated by the representative histological cross-sections showing medium-diameter test and control implants in Fig. 3a, b, respectively, all implants were well osseointegrated into bone. No evidence of fibrous encapsulation was histologically observed in any of the test or control specimens. As indicated by the study group, comparisons of BIC values in Fig. 3c and as supported by the descriptive statistics and statistical comparison in Tables 2 and 3, respectively, osseointegration of test and control implants was comparable for all tested implant diameter subgroups. Specifically, BIC values for the tests and control implants measured after 12 weeks of healing for the 3 subgroups of small-, medium-, and large-diameter implants were as follows: small-diameter implants (61.30 ± 10.63% vs. 54.46 ± 18.31%), medium-diameter implants (60.91 ± 14.42 vs. 54.68 ± 9.16) and large-diameter implants (45.60 ± 14.67 vs. 52.52 ± 14.76). Paired comparisons between test and control implants within the individual study subgroups of small-, medium-, and large-diameter implants by Wilcoxon rank tests resulted in *p* values of 0.99, 0.55, and 0.31 respectively, indicating that potential differences between test and control implants were not statistically significant. Also, from a qualitative perspective, the comparison of average BIC data of individual study subgroups did not reveal any major overall differences or trends.

**Table 2:** Descriptive statistics of histometric parameters

Group	Parameter	Test group	Control group
BIC (%)			
Small-diameter implants	*N*	8	7
	Mean ± SD	61.30 ± 10.63	54.46 ± 18.31
	Median (IQR)	59.12 (52.50 to 69.39)	55.29 (49.17 to 65.13)
Medium-diameter implants	*N*	8	8
	Mean ± SD	60.91 ± 14.42	54.68 ± 9.16
	Median (IQR)	63.92 (54.18 to 66.08)	53.84 (49.01 to 61.08)
Large-diameter implants	*N*	8	8
	Mean ± SD	45.60 ± 14.67	52.52 ± 14.76
	Median (IQR)	40.18 (34.78 to 56.29)	49.82 (40.44 to 62.50)
fBIC (μm)			
Small-diameter implants	*N*	8	7
	Mean ± SD	−19.01 ± 475.93	−1026.23 ± 1603.23
	Median (IQR)	−98.73 (−260.82 to 178.36)	−779.26 (−956.49 to 197.21)
Medium-diameter implants	*N*	8	8
	Mean ± SD	−136.85 ± 355.98	−376.35 ± 478.01
	Median (IQR)	−48.99 (−469.67 to 131.21)	−327.36 (−683.67 to −275.08)
Large-diameter implants	*N*	8	8
	Mean ± SD	−328.49 ± 379.04	−764.94 ± 363.48
	Median (IQR)	−192.67 (−660.77 to −27.13)	−841.36 (−995.39 to −567.85)

*fBIC*, first bone-to-implant contact; *BIC*, bone-to-implant contact; *N*, sample number; *SD*, standard deviation; *IQR*, interquartile range (from first to third quartile)

**Table 3:** Paired comparisons of outcomes measured in test and control implants

Parameter	Group	Difference: test-control	*p* value
		Mean diff ± SD of diff	Wilcoxon signed-rank test
BIC (%)	Small diameter	6.58 ± 18.87	0.99
	Medium diameter	6.23 ± 12.38	0.55
	Large diameter	−6.92 ± 16.07	0.31
fBIC (%)	Small diameter	1105.65 ± 1473.43	**0.047**
	Medium diameter	239.50 ± 473.27	0.25
	Large diameter	436.45 ± 716.70	0.078

*BIC*, bone-to-implant contact; *fBIC*, first bone-to-implant contact; *SD*, standard deviation; *diff*, difference

Bold indicates statistical significance

**Fig. 4:**
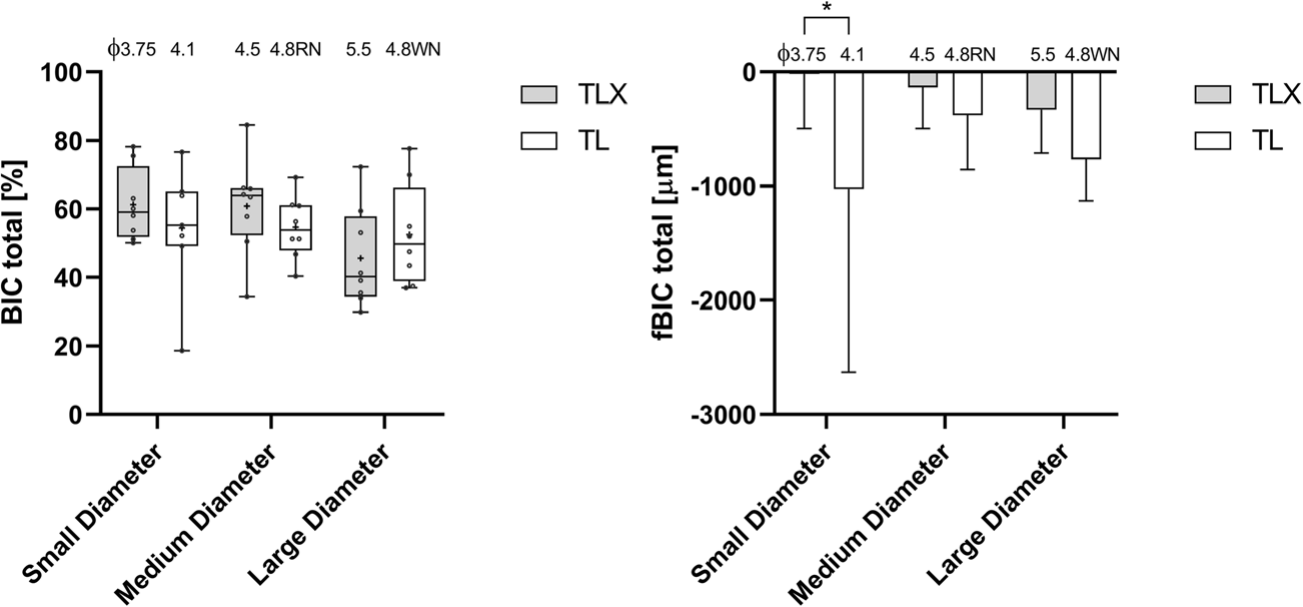
Left: Histometrically derived average total BIC per implant group. Right: Comparison of marginal bone loss between test and control implants per implant group as derived by the histometrically measured first bone-to-implant contacts (fBIC) and corresponding standard deviations. Box and whiskers plot of corresponding histometrically derived average total BIC per implant group. Boxes represent medians and upper (75%) and lower (25%) quartiles, average values are represented by crosses, open circles represent values of individual specimens, and whiskers demark the full data range from minimum to maximum registered individual values. Asterisk indicates a level of significance *p*≤0.05

The comparison of peri-implant crestal bone levels between test and control implants of specific diameters is represented in the plot of fBIC values in Fig. 4 and summarized in Table 2. Table 3 further summarizes the results of the paired statistical comparison as performed for the corresponding implant diameter subgroups. As evidenced by these comparisons, average fBIC values for test implants were consistently higher compared to the corresponding control implants. Specifically, small-diameter test implants resulted in fBIC values of −19.01 ± 475.93 μm compared to −1026.23 ± 1603.23 μm for the control implants. Results from the Wilcoxon signed-rank tests indicated statistical significance (*p*=0.047) (Table 3). Crestal bone levels and fBIC values for medium- and large-diameter test implants (−136.85 ± 355.98 μm and −328.49 ± 379.04 μm respectively) also tended to be higher compared to the corresponding values obtained for the control implants (−376.35 ± 478.01 μm and −764.94 ± 363.48 μm); however, differences within these study subgroups were not statistically significant (*p*= 0.25 and 0.078 respectively). As further evidenced by the plot of fBIC values in Fig. 4, test implants showed an apparent trend for lower fBIC values with increasing implant diameter. From a qualitative perspective, relatively high standard deviations were noted for the small-diameter control implants when compared to corresponding values of all other study groups.

### Statistical adjustment of histometric parameters and non-inferiority testing

In order to test for non-inferiority of osseointegration and crestal bone level evolution of test implants compared to control implants, mean values of BIC and fBIC were statistically adjusted and compared with regards to their pre-defined null and alternative hypothesis within their corresponding implant diameter subgroups. Table 4 summarizes the main parameters of the mixed linear models and associations between the corresponding histometric parameters and the study implants as well as the test parameters that were used for the non-inferiority testing, i.e., the average effects of the factor implant type and the tolerance ranges defining the 90% confidence intervals of the individual factors. The full set of results of the statistical analysis is provided in the supplementary part (Tables S1 and S2). As evidenced by the comparisons in Table 4, all calculated average effects for both parameters BIC and fBIC were well within the pre-defined corresponding confidence intervals. As a consequence, the null hypothesis was rejected and the alternative hypothesis was accepted for both parameters. This outcome statistically supports the observation that both the osseointegration as well as the crestal bone levels of the test implants were comparable and not statistically inferior to the corresponding control implants of the specific implant diameters.

**Table 4:** Adjusted^†^ association between histomorphometric outcomes and implant type and non-inferiority test for the implant of interest (*SE*, standard error; *CI*, confidence interval)

Parameter	Group	Regression model^†^		Adjusted means^†^			Non-Inferiority test	
		Regression estimate	SE	Mean	95% CI	*p* value^‡^	Average effect of the factor	(90% CI)^§^
Small-diameter implants								
BIC (%)	Test	2.55	5.81	60.60	46.95 to 74.25	0.6908	2.55	−11.13 to 16.22
	Control	0.00		58.05	44.08 to 72.02	Ref		
fBIC (%)	Test	646.47	463.95	416.49	−1506.36 to 673.38	0.2578	646.47	−445.38 to 1738.31
	Control	0.00		−1062.96	2178.45 to 52.54	-		
Medium-diameter implants								
BIC (%)	Test	1.031	2.53	57.30	47.09 to 67.52	0.7043	1.03	−4.36 to 6.42
	Control	0.00		56.27	46.24 to 66.31			
fBIC (%)	Test	119.47	194.85	−208.09	−649.12 to 232.94	0.5729	119.47	−295.92 to 534.85
	Control	0.00		327.56	−760.60 to 105.48			
Large-diameter implants								
BIC (%)	Test	−7.55	5.09	46.35	36.01 to 56.69	0.1985	−7.55	−17.81 to 2.72
	Control	0.000		53.90	44.26 to 63.53			
fBIC (%)	Test	414.79	203.07	−375.45	−776.67 to 25.77	0.0966	414.79	5.59 to 824.00
	Control	0.00		−790.24	−1164.70 to −415.79			

^†^Mixed linear models were used to estimate the association. The association was adjusted by the fixed effects mandible side and mesio-distal position as well as by the individual test animal as a random effect

^‡^Adjusted for multiple comparisons according to Dunnett-Hsu

^§^CI = Confidence Interval

### Simulated Insertion and insertion torque measurements

**Fig. 5:**
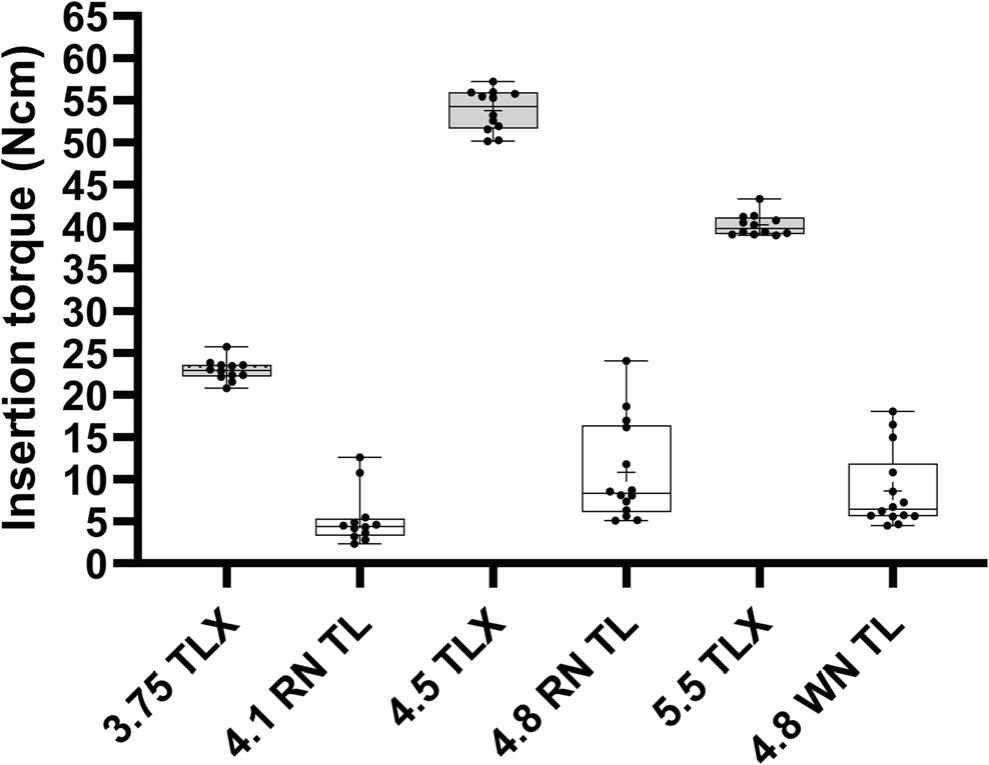
Maximum insertion torques values for test and control implants per tested implant group. Boxes represent medians and upper (75%) and lower (25%) quartiles, average values, and standard deviations are represented by crosses and whiskers. Plus sign = mean. Dots = individual data points

Average maximum insertion torques as derived during simulated insertion experiments in PU plates were consistently higher for test implants compared to control implants for all tested implant diameters (Fig. 5). Specifically, the average maximum insertion torque values of control implants ranged from 5.3 ± 3.1 Ncm for small-diameter implants to 10.8 ± 5.9 Ncm for medium-diameter implants. Corresponding values for the test implants, on the other hand, ranged from 23.0 ± 1.3 Ncm for the small-diameter implants to 53.8 ± 2.4 Ncm for the medium-diameter implants. Intermediate values were obtained for large-diameter implants with values of 8.7 ± 4.6 Ncm and 40.2 ± 1.3 Ncm for control and test implants, respectively. As evidenced by the box plots in Fig. 5 standard deviations were low compared to the differences between individual average values of the individual corresponding test and control groups.

## Discussion

From the comparison of the test and control implants, three main observations were obtained: (1) test and control implants resulted in equivalent and non-inferior levels of osseointegration (BIC) independent of the implant diameter, (2) crestal bone levels (fBIC) of test implants were non-inferior to control implants for all tested diameters. Values were higher for the test implants compared to the corresponding control implants with differences being statistically significant for the small-diameter implant group (3). Insertion torque values of all test implants were significantly higher when compared to implants of the corresponding control group.

The model system, implant groups, control groups, and surgical protocol were chosen with the aim of rendering the study as clinically meaningful as possible. Care was taken to strictly adhere to the implant manufacturer’s instructions during implant placement. The bone quality was a medium, type 2/3 bone quality in order to specifically challenge the novel implant design and associated surgical workflows under conditions with moderate bone quality. This bone quality might be specifically encountered in the primary clinical target indication of the investigated novel tapered implant [18]. More generally the physiologic and bone metabolic characteristics of porcine animal models have been described as very similar to the ones of humans and can be considered as well established for the investigation of osseointegration of dental implants as a function of implant design [19]. With regards to the investigation of crestal bone level changes, it is further important to exclude any changes that might be attributed to implant infection. In this study, no signs of peri-implant inflammation were evident at the time of termination. Therefore, it can be assumed that the observed crestal bone level differences between test and control implants were directly related to differences in implant design and placement protocols. With regards to the investigation of crestal bone levels, a fully healed bone situation was preferred over any early or immediate placement protocol to ensure a high level of standardization at baseline.

With regards to the histological and histometric outcomes of this study, four main potential influencing factors that varied between test and control implants need to be considered: (1) subcrestal implant design, (2) implant design at crestal level, (3) surgical protocols for osteotomy preparation, and (4) supracrestal implant design. Differences between both implant types were most pronounced with regards to the shape and design at intrabony and crestal level, while supracrestal design and dimensions were widely comparable. Pronounced differences between the osteotomies’ dimensions and contours in relation to implant shape and dimensions existed between test and control implants (see Table 1). Specifically, control implants were placed into diameter and contour matching osteotomies, while test implants were placed into parallel-walled osteotomies that were over and under-contoured with respect to the core diameter and thread dimensions of the implants, respectively. This aspect needs to be considered in the context of any potential influence of implant design on the histometric outcomes [20]. The chosen study design does not allow to differentiate between the influence of individual design features like implant shape, thread design, and osteotomy preparation on primary stability and osseointegration. However, the results are well in line with studies and summaries that have addressed and discussed the influence of such parameters individually and in more detail [13–15, 21–23]. Particularly within a recent pre-clinical study, Jimbo et al. have analyzed the combined effects of drilling regimens and implant macro-geometry on bone integration showing that both parameters had a significant effect not only on bone formation itself but also on the underlying biological processes [23]. This observation might be supported by qualitative differences in bone formation that were observed around the apical parts of the implants. Specifically, control implants were surrounded homogenously by bone, while bone contact to test implants appeared to be more associated with the threads of the implants. Despite these purely qualitative differences, bone integration in terms of BIC values was comparable for both implant types. The hypothesis of potentially different underlying biological mechanisms of bone integration might also be supported by the ability of test implants to cut, collect, and condense bone chips in the implant osteotomy when compared to control implants. This process might promote osseointegration in “healing chambers” as suggested by Jimbo et al. and might contribute to the observed equivalent levels of bone integration when compared to control implants [17, 23]. A further contributing factor to comparable BIC between the test and control implants could be related to the SLActive surface used in both groups. SLActive is reported to speed up the early osseointegration phase [24], perhaps enabling the test implant, regardless of the gap between the osteotomy and implant core, to promote rapid bone formation. While it was not the subject of the present study, one can imagine that the two phenomena (bone chip creation around the implant and the SLActive surface) worked in synergy to result in comparable BIC measures between the test and control implants at the given time points. Another important aspect in this context is the pronounced higher primary stability of the test implants compared to control implants. This higher primary stability and insertion torque of test implants does not only confirm the potential suitability of test implants for immediate placement but can also be considered as an indicator for the significant differences in implant thread design and osteotomy preparation between both tested implant types [15].

Another interesting observation of the study is related to the differences in crestal bone levels, which showed a constant trend for higher values for the test implants compared to control implants. Differences were most pronounced for small-diameter implants. Possible effects might again be associated with implant design, specifically neck design, and osteotomy preparation or a combination thereof. On a clinical level, studies that clearly indicate an influence of the implant type and specifically implant design on marginal bone levels are scarce and conclusions from such studies remain inconclusive [25, 26]. However, pre-clinical studies clearly indicate an influence of both implant neck design and surgical techniques on crestal bone level preservation [20, 27–29].

In order to achieve high levels of primary stability, one of the strategies is to under-prepare the implant osteotomy with regards to the implant diameter. Although this strategy has proven effective in interlocking the implant with cortical bone there is also a moderate risk of inducing crestal bone level changes caused by a compression of the crestal bone [13, 20, 21]. For both investigated implant types, under-preparation of the osteotomy with regards to the implant diameter was excluded. Therefore, differences in marginal bone levels might more likely be associated with differences in implant design at crestal level and with differences in the height and curvature of the implant neck at supracrestal level or a combination thereof. Specifically, the implant neck at crestal level of control implants displayed a divergent diameter, while test implants were slightly concave shaped at crestal level and displayed a reduced diameter to minimize potential stresses on the cortical bone [17]. Cohen et al. have recently shown in a pre-clinical study that this strategy of under-contouring the osteotomy with regards to the implant neck diameter might lead to superior crestal bone levels. Another apparent difference is related to the horizontal offset between the apical platform of the implants extending over the implant core, which generates an umbrella-like arrangement over the soft tissues and crestal bone levels. Compared to the control implants, this offset was higher for the test implants and was interestingly most pronounced for the large-diameter implants. The latter displayed an offset of 1 mm per side combined with a relative short and curved implant neck when compared to small- and medium-diameter implants. The potential effect that the specific supracrestal implant shapes and designs exert on crestal bone level preservation might motivate future studies that address this aspect in more detail.

Finally, a couple of limitations should be mentioned with the present study. Firstly, the test implant is more appropriate in an immediate placement indication due to its fully tapered body, wide threads, and high insertion torque profile. The TLX implant was tested in a healed site, perhaps not representing the full clinical scenario of such an implant but providing instead important data on the osseointegration behavior as compared to a control device in a standardized osteotomy model. Secondly, fBIC (bone height changes) are reported at 3 months. This is technically a rather early healing time point. However, a previous study reported significant fBIC changes at 3 months in the same minipig model when comparing different osteotomy production protocols [30]. It is therefore reasonable to expect that if a complication resulting in a negatively affected fBIC were to occur, 3 months is sufficient to capture this phenomenon.

## Conclusion

Both implant types displayed non-inferior and equivalent levels of osseointegration and bone height maintenance, while the test implants displayed significantly higher primary implant stability. The combination of observations indicates that the novel implant type is able to provide high levels of primary combined with a comparable osseointegration pattern to benchmark TL implants.

## Supplementary Information

ESM 1 (DOCX 29 kb).
