# Supplementary material for: A novel fully tapered, self-cutting tissue-level implant: non-inferiority study in minipigs
Source: Clin Oral Investig. 2021 Apr 16;25(11):6127–37. doi: 10.1007/s00784-021-03912-w (PMC8531107; doi:10.1007/s00784-021-03912-w)
Supplement: Supplementary file 1 — (DOCX 29 kb). [file 784_2021_3912_MOESM1_ESM.docx]

# Supplementary data

## Statistically adjusted data sets and non-inferiority testing

To test non-inferiority of test compared to control implants corresponding histomorphometric parameters BIC and fBIC were statistically adjusted for potential effects of the individual animal, side of the jaw and implant position (anterior, middle, posterior) using a multivariable mixed linear regression model. The full set of results from these models are shown for the individual implant diameter groups of small, medium and large implants in Table S1 and S2 respectively.

## Non-inferiority testing of BIC values

Specifically adjusted average BIC of test implants and control small diameter implants were 60.60% and 58.05% respectively with a Dunnett-Hsu p-value of 0.6908. The average effect of the factor (the mean difference between the implants) was determined to be 2.55% compared to an acceptable tolerated difference of -11.13% that would have been required to reject the hypothesis of non-inferiority.

Likewise, average BIC of medium diameter test and control implants were 57.30% and 56.27% respectively with a Dunnett-Hsu p-value of 0.7043. The average effect of the factor implant type was 1.03% which was higher compared to the minimum threshold of -4.36% that would have been required to reject the hypothesis of non-inferiority.

Finally, average BIC values of large diameter test and control implants were 46.35% and 53.90% respectively (Dunnett-Hsu p-value=0.1985). The average effect of the factor was determined to be -7.55%. This average effect was higher compared to the minimum threshold of -17.81% that was determined to be necessary to reject the hypothesis of non-inferiority of this implant type.

Concluding non-inferiority testing of the average BIC values by means of multivariable mixed regression models resulted in the conclusion that test implants were non-inferior when compared to control implants for all three implant diameter groups.

## Non-inferiority testing of fBIC values

Adjusted average fBIC of test implants and control small diameter implants were -416.49µm and -1062.96µm respectively (Dunnett-Hsu p-value = 0.2578). The average effect of the factor implant type (the mean difference between the implants) on the total fBIC was determined to be 646.47µm. The minimum acceptable tolerated average effect to support the null hypothesis was -445.38µm. As a result, the null-hypothesis was rejected and the hypothesis of non-inferiority of test and control implants was supported.

Likewise average fBIC of medium diameter test and control implants were -208.09µm and
-327.56µm respectively with a Dunnett-Hsu p-value of 0.5729. The average effect of the factor implant type was 119.47µm which was higher compared to the minimum threshold of
-295.92µm for acceptance of the null-hypothesis. As a result, the null-hypothesis was rejected and the alternative hypothesis of non-inferiority of control and test implants was supported by the test.

Finally, average fBIC values of large diameter test and control implants were -375.45µm and
-790.24µm respectively (Dunnett-Hsu p-value=0.0966). The average effect of the factor was determined to be 414.79µm. Again this average effect was higher compared to the minimum threshold of –824.00µm for acceptance of the null hypothesis. Consequently, also for large diameter implants the null-hypothesis was rejected and the alternative hypothesis of non-inferiority was supported.

Concluding non-inferiority testing of the average fBIC values by means of multivariable mixed regression models resulted in the conclusion that test implants were non-inferior when compared to control implants for all three tested implant diameter groups.

Table S1: Complete set of BIC values and derived parameters used for the non-inferiority comparison of test to control devices for the three subgroups of small, medium and large diameters adjusted† for the factors side, position and animal effects. The effect of the animal was introduced as a random effect. P-value were adjusted for multiple comparisons using the Dunnet-Hsu test method.

| Outcome | Factor | Value | Regression parameters | | | Adjusted parameters for multiple comparisons^§^ | | | Non-Inferiority | |
| --- | --- | --- | --- | --- | --- | --- | --- | --- | --- | --- |
|  |  |  | Estimate | SE | (t-Test)_Reg_  p-Value | Adjusted mean | 95% CI for the adjusted mean | Dunnett-Hsu  p-Value | Average effect of the factor | (90% CI) ^§§^ |
| Small Diameter group  BIC total [%] | Intercept |  | 49.510 | 6.938 | 0.0002 |  |  |  |  |  |
|  | Test item | Test Implant | 2.547 | 5.810 | 0.6908 | 60.60 | 46.95 - 74.25 | 0.6908 | 2.55 | -11.13 - 16.22 |
|  |  | Control Implant | 0.000 |  |  | 58.05 | 44.08 - 72.02 | *Ref.* |  |  |
|  | Position | anterior | 10.624 | 7.682 | 0.2606 | 56.84 | 44.77 - 68.90 | 0.3844 |  |  |
|  |  | posterior | 28.717 | 9.111 | 0.0512 | 74.93 | 54.46 - 95.40 | 0.0803 |  |  |
|  |  | middle | 0.000 |  |  | 46.21 | 25.65 - 66.78 | *Ref.* |  |  |
|  | Side | Left | -9.145 | 6.001 | 0.2249 | 54.75 | 40.05 - 69.45 | 0.2249 |  |  |
|  |  | Right | 0.000 |  |  | 63.90 | 50.60 - 77.19 |  |  |  |
| Medium Diameter group  BIC total [%] | Intercept |  | 58.840 | 4.549 | <.0001 |  |  |  |  |  |
|  | Test item | Test Implant | 1.031 | 2.529 | 0.7043 | 57.30 | 47.09 - 67.52 | 0.7043 | 1.03 | -4.36 - 6.42 |
|  |  | Control Implant | 0.000 |  |  | 56.27 | 46.24 - 66.31 |  |  |  |
|  | Position | Anterior | 3.840 | 6.699 | 0.5972 | 65.64 | 49.43 - 81.84 | 0.7998 |  |  |
|  |  | posterior | -18.872 | 5.266 | 0.0231 | 42.93 | 29.85 - 56.00 | 0.0389 |  |  |
|  |  | Middle | 0.000 |  |  | 61.80 | 51.00 - 72.60 |  |  |  |
|  | Side | Left | 4.883 | 2.408 | 0.1125 | 59.23 | 48.78 - 69.67 | 0.1125 |  |  |
|  |  | Right | 0.000 |  |  | 54.35 | 44.67 - 64.02 |  |  |  |
| Large Diameter group  BIC total [%] | Intercept |  | 74.305 | 6.417 | <.0001 |  |  |  |  |  |
|  | Test item | Test Implant | -7.548 | 5.094 | 0.1985 | 46.35 | 36.01 - 56.69 | 0.1985 | -7.55 | -17.81 - 2.72 |
|  |  | Control Implant | 0.000 |  |  | 53.90 | 44.26 - 63.53 |  |  |  |
|  | Position | anterior | -26.509 | 8.121 | 0.0223 | 39.62 | 23.74 - 55.50 | 0.0382 |  |  |
|  |  | posterior | -21.510 | 6.346 | 0.0195 | 44.62 | 35.54 - 53.70 | 0.0334 |  |  |
|  |  | middle | 0.000 |  |  | 66.13 | 52.58 - 79.68 |  |  |  |
|  | Side | Left | -8.804 | 5.094 | 0.1445 | 45.72 | 35.38 - 56.06 | 0.1445 |  |  |
|  |  | Right | 0.000 |  |  | 54.52 | 44.89 - 64.16 |  |  |  |

^§^The factor animal was introduced in the model as a random effect

^§§^90%CI for an alpha=0.10 two tailed which is equivalent to a 95%for an alpha of 0,05 one tailed

Ref. = Reference level for the comparison within a factor.

Table S.2: Complete set of fBIC values and derived parameters used for the non-inferiority comparison of test to control devices for the three subgroups of small, medium and large diameters adjusted† for the factors side, position and animal effects. The effect of the animal was introduced as a random effect. P-value were adjusted for multiple comparisons using the Dunnet-Hsu test method.

| Outcome | Factor | Value | Regression parameters | | | Adjusted parameters for multiple comparisons^§^ | | | Non-Inferiority | |
| --- | --- | --- | --- | --- | --- | --- | --- | --- | --- | --- |
|  |  |  | Estimate | SE | (t-Test)_Reg_  p-Value | Adjusted mean | 95% CI for the adjusted mean | Dunnett-Hsu  p-Value | Average effect of the factor | (90% CI) ^§§^ |
| Small Diameter group  fBIC total [µm] | Intercept |  | -1683.801 | 554.010 | 0.0189 |  |  |  |  |  |
|  | Test item | Test Implant | 646.465 | 463.950 | 0.2578 | -416.49 | -1506.36 - 673.38 | 0.2578 | 646.47 | -445.38 - 1738.31 |
|  |  | Control Implant | 0.000 |  |  | -1062.96 | -2178.45 - 52.54 |  |  |  |
|  | Position | Anterior | 1847.410 | 613.420 | 0.0571 | -73.52 | -1037.12 - 890.09 | 0.0894 |  |  |
|  |  | posterior | 1696.205 | 727.570 | 0.102 | -224.72 | -1859.52 - 1410.08 | 0.1574 |  |  |
|  |  | Middle | 0.000 |  |  | -1920.93 | -3563.12 - -278.74 |  |  |  |
|  | Side | Left | -1120.719 | 479.170 | 0.1013 | -1300.08 | -2473.78 - -126.39 | 0.1013 |  |  |
|  |  | Right | 0.000 |  |  | -179.36 | -1241.09 - 882.36 |  |  |  |
| Medium Diameter group  fBIC total [µm] | Intercept |  | -275.797 | 212.740 | 0.2359 |  |  |  |  |  |
|  | Test item | Test Implant | 119.467 | 194.850 | 0.5729 | -208.09 | -649.12 - 232.94 | 0.5729 | 119.47 | -295.92 - 534.85 |
|  |  | Control Implant | 0.000 |  |  | -327.56 | -760.60 - 105.48 |  |  |  |
|  | Position | Anterior | 153.538 | 294.890 | 0.6301 | -31.01 | -739.09 - 677.07 | 0.843 |  |  |
|  |  | posterior | -403.377 | 264.060 | 0.2013 | -587.92 | -1198.51 - 22.67 | 0.3308 |  |  |
|  |  | Middle | 0.000 |  |  | -184.55 | -595.71 - 226.62 |  |  |  |
|  | Side | Left | 63.035 | 187.120 | 0.7531 | -236.31 | -682.80 - 210.18 | 0.7531 |  |  |
|  |  | Right | 0.000 |  |  | -299.34 | -713.23 - 114.54 |  |  |  |
| Large Diameter group  fBIC total [µm] | Intercept |  | -737.295 | 247.530 | 0.0247 |  |  |  |  |  |
|  | Test item | Test Implant | 414.794 | 203.070 | 0.0966 | -375.45 | -776.67 - 25.77 | 0.0966 | 414.79 | 5.59 - 824.00 |
|  |  | Control Implant | 0.000 |  |  | -790.24 | -1164.70 - -415.79 |  |  |  |
|  | Position | anterior | -42.836 | 309.570 | 0.8953 | -654.86 | -1261.93 - -47.80 | 0.9858 |  |  |
|  |  | posterior | 130.382 | 241.100 | 0.6119 | -481.64 | -827.16 to - 136.13 | 0.8155 |  |  |
|  |  | middle | 0.000 |  |  | -612.03 | -1126.53 - -97.52 |  |  |  |
|  | Side | Left | -164.255 | 203.070 | 0.4554 | -664.97 | -1066.19 - -263.75 | 0.4554 |  |  |
|  |  | Right | 0.000 |  |  | -500.72 | -875.17 - -126.26 |  |  |  |

^§^The factor animal was introduced in the model as a random effect

^§§^90%CI for an alpha=0.10 two tailed which is equivalent to a 95%for an alpha of 0,05 one tailed

Ref. = Reference level for the comparison within a factor.
